# Sharing of *cmeRABC* alleles between *C. coli* and *C. jejuni* associated with extensive drug resistance in *Campylobacter* isolates from infants and poultry in the Peruvian Amazon

**DOI:** 10.1128/mbio.02054-24

**Published:** 2024-12-27

**Authors:** Kerry K. Cooper, Evangelos Mourkas, Francesca Schiaffino, Craig T. Parker, Tackeshy N. Pinedo Vasquez, Paul F. Garcia Bardales, Pablo Peñataro Yori, Maribel Paredes Olortegui, Katia Manzanares Villanueva, Lucero Romaina Cachique, Hermann Silva Delgado, Matthew D. Hitchings, Steven Huynh, Samuel K. Sheppard, Ben Pascoe, Margaret N. Kosek

**Affiliations:** 1 School of Animal and Comparative Biomedical Sciences, University of Arizona, Tucson, Arizona, USA; 2 Zoonosis Science Center, Department of Medical Sciences, Uppsala University, Uppsala, Sweden; 3 Division of Infectious Diseases and International Health, School of Medicine, University of Virginia, Charlottsville, Virginia, USA; 4 Faculty of Veterinary Medicine, Universidad Peruana Cayetano Heredia, San Martin de Porres, Lima, Peru; 5 Produce Safety and Microbiology Research Unit, Agricultural Research Service, U.S. Department of Agriculture, Albany, California, USA; 6 Asociacion Benefica Prisma, Iquitos, Peru; 7 School of Human Medicine, Universidad Nacional de la Amazonia Peruana, Iquitos, Peru; 8 Swansea University Medical School, Swansea University 7759 , Swansea, United Kingdom; 9 Ineos Oxford Institute for Antimicrobial Research, Department of Biology, University of Oxford, Oxford, United Kingdom; LMU Munich, Munich, Germany

**Keywords:** *Campylobacter*, efflux pump, antibiotic resistance, recombination, Iquitos

## Abstract

*Campylobacter* is a serious health threat because of the rapid progressive evolution of antimicrobial resistance and efficient transmission from zoonotic as well as human sources. Resistance to fluoroquinolones and macrolides is particularly concerning as this compromises the two most effective oral antibiotic agents currently available for human campylobacteriosis. Here, we report on the prevalence and worldwide distribution of the operon *cmeRABC*, which encodes an efflux pump conferring high levels of combined resistance to fluoroquinolones and macrolides in *Campylobacter* strains isolated from poultry (*n* = 75) and children (*n* = 177). These mutations were found to be highly prevalent in isolates from poultry (62.7%) and children (29.4%) in Iquitos, Peru. We investigated the population structure of genes in the *cmeRABC* operon and identified a potential genetic bottleneck for the *cmeA* and *cmeB* genes. While most *cmeB* alleles segregate by species, alleles associated with high resistance to fluoroquinolones and macrolides were found in both *Campylobacter jejuni* and *Campylobacter coli*. We inferred that the likely ancestry of these alleles was from *C. jejuni* and was later acquired by *C. coli* through recombination. Publicly accessible global genomic data from 16,120 *Campylobacter* genomes identified these mutations in approximately 6% of *C. jejuni* and *C. coli* isolates globally, with higher prevalence in samples from poultry in many countries, including Peru. Our findings suggest that these extensively drug-resistant *Campylobacter* strains originated from *C. jejuni* in poultry.

**IMPORTANCE:**

Antimicrobial resistance in *Campylobacter* is a growing public health concern, driven by the rapid evolution and zoonotic transmission of resistant strains. This study focuses on mutations in the cmeABC efflux pump, which confer high resistance to fluoroquinolones and macrolides, the two most effective oral antibiotics for human campylobacteriosis. By analyzing genomes from poultry and children in Iquitos, Peru, as well as global genomic data sets, we identified a significant prevalence of these resistance-associated mutations, particularly in poultry and children. Our findings suggest that these mutations originated in *Campylobacter jejuni* and spread to *C. coli* through recombination. Globally, these mutations are found in approximately 6% of isolates, with higher prevalence in poultry in multiple countries. This research underscores the critical role of genomic epidemiology in understanding the origins, evolution, and dissemination of antimicrobial resistance and highlights the need to address poultry as a reservoir for resistant *Campylobacter*.

## INTRODUCTION

*Campylobacter is* the leading cause of bacterial gastroenteritis worldwide (1–3). Rising global trends in multidrug-resistant (MDR) *Campylobacter jejuni* and *Campylobacter coli coli* represent a serious public health risk (4). Fluoroquinolone-resistant strains are identified as a serious threat by both the United States Centers for Disease Control (5) and the World Health Organization (6). Consequently, azithromycin (a macrolide) has become the primary treatment for acute gastroenteritis caused by *Campylobacter* (7–9).

Increased antimicrobial resistance (AMR) in *Campylobacter* is posited as the consequence of the extensive use of antimicrobials in livestock animals (10, 11), particularly in countries where antibiotics are commonly employed as growth promoters (12). In *Campylobacter*, resistances to fluoroquinolones and macrolides primarily arise from specific chromosomal mutations. Quinolone resistance results from a mutation in the *gyrA* gene (13) with no associated fitness costs. Macrolide resistance is typically due to mutations in the 23S rRNA gene or the acquisition of the *ermB* gene (14–17). Additionally, the CmeABC efflux system, a tripartite drug efflux pump, has been demonstrated to play a pivotal role in transporting antimicrobials out of *Campylobacter* cells, resulting in high-level resistance to diverse classes of antimicrobials (18–20).

Resistance–nodulation–cell division (RND) efflux systems, such as CmeABC, enhance resistance to bile salts and synergize with other resistance determinants, contributing to increased resistance to various antimicrobials (19–22). The system is encoded by the *cmeABC* operon located in the chromosome of the bacteria. It encodes a periplasmic fusion protein (CmeA), an inner membrane transporter (CmeB), and an outer membrane protein (CmeC) (20, 22, 23). The *cmeABC* operon is regulated by the transcriptional repressor CmeR, which binds to the inverted repeats on the promoter region of the operon, where even single-base changes can alter CmeR binding, resulting in overexpression of the *cmeABC* operon (24). In *Campylobacter*, the expression of the *cmeABC* efflux pump genes is elevated in the presence of bile salts, making it crucial for bacterial intestinal colonization and survival (25, 26). This efflux system is also linked to resistance to heavy metals and disinfectants (20, 26). A multidrug-resistant variant of the *cmeABC* operon, known as RE-*cmeABC*, has been identified in *C. jejuni* isolates globally, including in Peru, where it is strongly associated with multidrug-resistant profiles (19, 21, 27, 28).

Given the widespread resistance across antibiotic classes (27, 29) and enhanced resistance associated with RE-*cmeABC* (27), we conducted a study to (i) further characterize the RE-*cmeABC* genotype in Peruvian isolates derived from both poultry and humans; (ii) determine the distribution and composition of *cmeB* alleles among *C. jejuni* and *C. coli* strains in Peru; and (iii) determine the distribution of RE-*cmeABC* alleles in a publicly available global collection of *Campylobacter* genomes.

## MATERIALS AND METHODS

### Sample collection and processing

Human *C. jejuni* and *C. coli* isolates were derived from three studies conducted in Iquitos, Peru. Two of these were birth cohorts occurring between 2009 and 2024. Sampling criteria for both cohorts have been described previously (30, 31). Specifically, children were enrolled within 17 days of birth and followed up continuously for up to 5 years for the first cohort (study A) and 2 years for the second (study B). Stool samples were collected every time a child experienced diarrhea, and monthly specimens were collected for surveillance purposes. For study B, in case *Campylobacter* was detected by standard microbiology during an episode of diarrhea, children were enrolled into a nested case–control study enrolling an age-matched child. All children and adults who provided consent, as well as all animals living within the household premise of the case and control households, were also sampled for *Campylobacter* within 5 days of culture positivity of the index child. The third study (study C) was conducted between 2019 and 2022 and enrolled children under the age of 2 years who sought care for an episode of diarrhea at local primary and tertiary care centers in Iquitos, Peru. All fecal samples were collected using a sterile cotton swab directly from a diaper or a plastic container and placed directly in a Cary Blair transport medium. Samples were processed and cultured within 12 h of collection.

Poultry fecal samples were obtained from two distinct sampling events. The first one (study D) took place between August and December 2019. Households in Santa Clara de Nanay (a periurban community in Iquitos, Loreto, Peru) were surveyed for the presence of chickens by local field workers. Specifically, households that had chickens in their backyards (“chacras”) or inside the living domain of the household were identified by asking the household owner. Of these, a random sample was selected, and between three and five fecal samples from backyard poultry (crossbreed, *Gallus gallus*) were collected per household. Additionally, fecal samples from commercially raised broilers (White Leghorn or Cornish, *Gallus gallus*) were collected from two live poultry markets located at Iquitos city center within the same time range. The second sampling event was part of the nested case–control study described previously (study B), in which all animals from case and control households were sampled. All poultry fecal samples were collected using a sterile cotton swab as soon as the bird voided and placed directly in a Cary Blair transport medium. Samples were processed and cultured within 12 h of collection.

### *Campylobacter* culture and identification

Human fecal samples from the first cohort, as well as poultry fecal samples, were cultured using *Campylobacter* Blood-Free Selective Agar Base (Oxoid; Thermo Fisher Scientific, Waltham, MA) with CCDA Selective Supplement (Oxoid, Thermo Fisher Scientific), at 42°C in microaerophilic (1% O_2_ + 10% CO_2_ + 10% H_2_ + balance N_2_) conditions. All other human fecal samples were cultured using Columbia Blood Agar Base (Oxoid, Thermo Fisher Scientific) supplemented with 5% lysed horse blood and an S-pack filter of 0.45 μM and 47 mm in diameter (Merck Millipore, Burlington, MA), in microaerophilic conditions at 37°C. Colonies with compatible *Campylobacter* morphology were further confirmed as *Campylobacter* spp. or *C. jejuni*/*C. coli* using a duplex quantitative PCR targeting the 16S rRNA and the *Campylobacter* adhesion to fibronectin (*cadF*) genes (32). Phenotypic antimicrobial susceptibility testing was performed using standard disk-diffusion testing (29) against the following antibiotics: ciprofloxacin (CIP), erythromycin (ERY), azithromycin (AZM), tetracycline (TET), gentamicin (GEN), amoxicillin and clavulanic acid (AMC), ampicillin (AMP), chloramphenicol (CHL), and imipenem (IMP). Zone diameter breakpoints (in millimeter) for *Campylobacter* spp. from the Clinical and Laboratory Standards Institute (CLSI) (CLSI M45) were applied to assess CIP, ERY, AZM, and TET resistance. The CLSI zone diameter breakpoints (mm) for Enterobacteriaceae were used for GEN, AMC, AMP, CHL, and IMP,.

### Whole-genome sequencing and genome archiving

Sequencing of genomic DNA from *Campylobacter* isolates from the first pediatric cohort was described previously (27, 33). For additional samples, libraries were prepared using the Illumina DNA Prep Tagmentation kit following the manufacturer’s instructions with the following changes to increase insert length: decreasing the first and second volumes of sample purification beads to 40 and 11 µL, respectively, and a final elution in 10 µL Illumina resuspension buffer. Illumina-DNA/RNA UD Plates A, B, C, and D dual index adapters were ordered from Integrated DNA Technologies (Coralville, IA) and used at 1 µM final concentration. Individual libraries were quantified using the KAPA Library Quantification Kit (Roche) in 10 µL volume reactions and 90 s annealing/extension PCR, pooled and normalized to 4 nM. Pooled libraries were requantified by droplet digital PCR (ddPCR) on a QX200 system (Bio-Rad, Hercules, CA), using the Illumina TruSeq ddPCR Library Quantification Kit following the manufacturer’s protocols. Libraries were sequenced using a MiSeq Reagent Kit (v.2) (500 cycles) on a MiSeq instrument (Illumina) at 16 pM, following the manufacturer’s protocols. Short-read data are available at the National Center for Biotechnology Information (NCBI) Sequence Read Archive with the accession numbers SRR28536576 through SRR28536596 and are also associated with BioProject PRJNA912682.

All genomes were assembled using the Spades assembler plugin for Geneious Prime (v.2023.2.1) (https://www.geneious.com) (34, 35). All assembled genomes were assessed using CheckM software (v.1.1.3) for completeness, contamination, and heterogeneity (36). All samples with contamination greater than 4% and heterogeneity greater than 50% were further analyzed using the Kraken taxonomic sequence classification System (37, 38). Samples in which bacterial genomes other than *Campylobacter* spp. were detected, and samples in which more than one *Campylobacter* spp. were detected were excluded from the analysis as definitive high-resolution typing was unable to be done. All strain metadata and sequencing statistics are available in Table S1.

### Core genome characterization

Multilocus sequence types (STs) and associated clonal complexes (CCs) were automatically determined using the PubMLST database (39, 40). The entire genome was submitted for the seven-gene multilocus sequence typing profiling for each of the isolates (41). The core, accessory, and pangenome were characterized using Roary (v.3.12.0) (42) at 90% identity. The total pangenome of the 252 *Campylobacter* strains used in this study was composed of 4,672 genes including 468 core genes (present in >99% of strains), 180 soft core genes (present in 95%–99% of strains), and 4,024 accessory genes. The 468 core genes were aligned using MAFFT (v.7.475) (43), and the best fit model for the alignment was determined using ModelTest-NG software (44, 45). A maximum likelihood tree was generated using RAxML (v.8.2.12) (46) with the general time reversible model with optimization of substitution rates, gamma distributed rates, and estimate of proportion of invariable sites with 1,000 bootstraps. The majority rule tree was visualized and *C. jejuni* (https://microreact.org/project/cYFcfdwgWY4JbH36B7NsGG-cooper-et-al-2024-cmerabc-jejuni-view) and *C. coli* (https://microreact.org/project/grEwxG5A7vv5kAeJRtJFyu-cooperetal-2024-cmerabc-coliview) phylogenies were displayed separately using Microreact (47).

To assess how each of the *Campylobacter* strains was related to each other based on the genes of the pangenome and the presence of either RE-CmeB or CmeB, a principal component analysis (PCA) plot based on a panmatrix was generated. To measure the distance between all the protein sequences in the pangenome and to generate the panmatrix, all the protein sequences of the strains were compared against each other using the blastAllAll, bDist, and panMatrix commands in the program Micropan (v.2.1) (48) in R. Finally, the PCA plot was generated using the panPCA command in the program and visualized using ggplot2.

### AMR genotyping

Genomes were mined for antimicrobial resistance chromosomal point mutations and antibiotic resistance genes using the Comprehensive Antibiotic Resistance Database, ResFinder and PointFinder, and NCBI AMRFinder databases (49–52). A positive match was determined when a gene had more than 80% nucleotide identity and more than 60% coverage. Mutations in the *cmeABC* efflux system operon were determined using BLASTN plugin in Geneious Prime (v.2022.2.2) (https://www.geneious.com). *cmeB* alleles that were below 84% nucleotide identity to *cmeB* of *C. jejuni* strain NCTC 11168 (accession number AL111168.1) and greater than 90% nucleotide identity to *cmeB* of *C. coli* strain DH161 (accession number KT778508.1) are hereby termed RE-*cmeB*.

### Genetic ancestry comparison of cmeRABC operon with other core genes

Single-gene phylogenies were constructed for each protein of the *cmeRABC* locus. The translated amino acid sequence was extracted from the genome of each isolate, for each gene individually, using Geneious Prime (v.2022.2.2, https://www.geneious.com). The 252 protein sequences for each gene were aligned against each other using MUSCLE (v.5.1) (53) with the default parameters and using ModelTest-NG software to determine the best-fit model for each of the four protein alignments. Maximum likelihood trees for the protein sequences for CmeR, CmeA, CmeB, and CmeC were generated using RAxML with an amino acid-specified matrix with a gamma model rate of heterogeneity, the estimated proportion of invariable sites, and the CPREV protein substitution model including 1,000 bootstraps. The majority rule tree for each of the proteins was visualized, and branches were colored in iTOL (v.6.6) (54).

The consistency of the phylogenetic tree with patterns of variation in sequence alignments for each gene of interest was calculated (55, 56). Consistency indices (CIs) were constructed for each single-gene alignment of *cmeABC* operon genes to a phylogeny using an alignment of 468 core genes shared by all isolates using the CI function of the R Phangorn package (57). The relative number of substitutions introduced by recombination and mutation was calculated as the ratio of recombination to mutation (*r*/*m*) for every branch and tip on the phylogeny reconstructed from the core genome using Gubbins (v.2.4.1) (58).

### Identification of populations of cmeB alleles using STRUCTURE

Most alleles in the *cmeRABC* operon are segregated by *Campylobacter* spp., of which some alleles are associated with high resistance to fluoroquinolones and macrolides. To quantify the segregation by population, a training data set was used to assign resistant alleles to the two *Campylobacter* spp., and the probability of predicting the correct species for each isolate (self-attribution) was recorded. Allele attribution was performed using STRUCTURE (59, 60), a Bayesian model-based clustering tool developed to infer population structure and attribute individuals to populations using genotype data. Probabilistic assignment was carried out using the “no admixture” model with uncorrelated allele frequency, assuming that each locus originated from one of the putative source populations, each with its own set of allelic frequencies. Analyses were performed with 50,000 burn-in cycles to ensure model stability, followed by 50,000 iterations with the parameters using source population information (USEPOPINFO), and test isolates were distinguished from the training data set using POPFLAG. For the training data, we chose random subsets of 18 isolates from each species and performed self-attribution 10 times to ensure consistency. The average probability of allele assignment to the correct *Campylobacter* spp. was used as a quantifiable measure of *cmeB* allele segregation by species.

### Global comparison of *cmeRABC* operon

PubMLST is a curated database (40) that contains >83,000 *Campylobacter* genomes from 9 *Campylobacter* spp., collected from more than 80 countries since 1970 (database accessed on 15 November 2023). Results were filtered for genomes that had metadata that included country, year of isolation, and host species. To determine which of the filtered genomes contained the RE-*cmeB* gene, BLAST searches were conducted to identify *cmeB* gene sequences as described above in the AMR Genotyping section. In total, 16,120 genomes with either the RE-*cmeB* gene or the normal *cmeB* gene were used for global distribution analysis. Barplots analyzing the frequency of the RE-*cmeB* gene based on *C. coli* clonal complex, *C. jejuni* clonal complex, year of isolation, poultry versus human, or other sources were generated using R (v.4.1.2) with the plyr and ggplot packages. The global distribution map of RE-*cmeB* gene by country was created using the chloroplethr package in R.

## RESULTS

A total of 252 *Campylobacter* isolates were sequenced, of which 160 were *C. jejuni* (105 human isolates and 55 poultry isolates) and 92 were *C. coli* (72 human isolates and 20 poultry isolates). Genome sequences were typed using the scheme from PubMLST (https://pubmlst.org/organisms/campylobacter-jejunicoli) (40, 41). The sizes of *C. jejuni* and *C. coli* genomes ranged between 1.58 and 1.94 Mb, with an average size of 1.74 Mb. The number of contigs ranged between 14 and 295, with a mean contig size of 30,378 bp per genome assembly. Details of the isolates, genomes, antibiotic resistance phenotypes, antibiotic resistance determinants, and genome quality are presented in Table S1. In total, 62.7% (47 of 75) of the poultry-associated *Campylobacter* genomes contained a *gyrA* mutation for ciprofloxacin resistance; 22.7% (17 of 75) had the 23S rRNA mutation for macrolide resistance; and 62.7% (47 of 75) had the RE*-cmeB* genotype, compared to 65.0% (115 of 177) of human-associated *Campylobacter* genomes that had the *gyrA* mutation, 22.0% (39 of 177) with the 23S rRNA mutation, and 29.4% (52 of 177) with the RE*-cmeB* genotype (Table S1).

### The high-resistance RE*-cmeB* genotype is dispersed among multiple *Campylobacter* lineages

**Fig 1:**
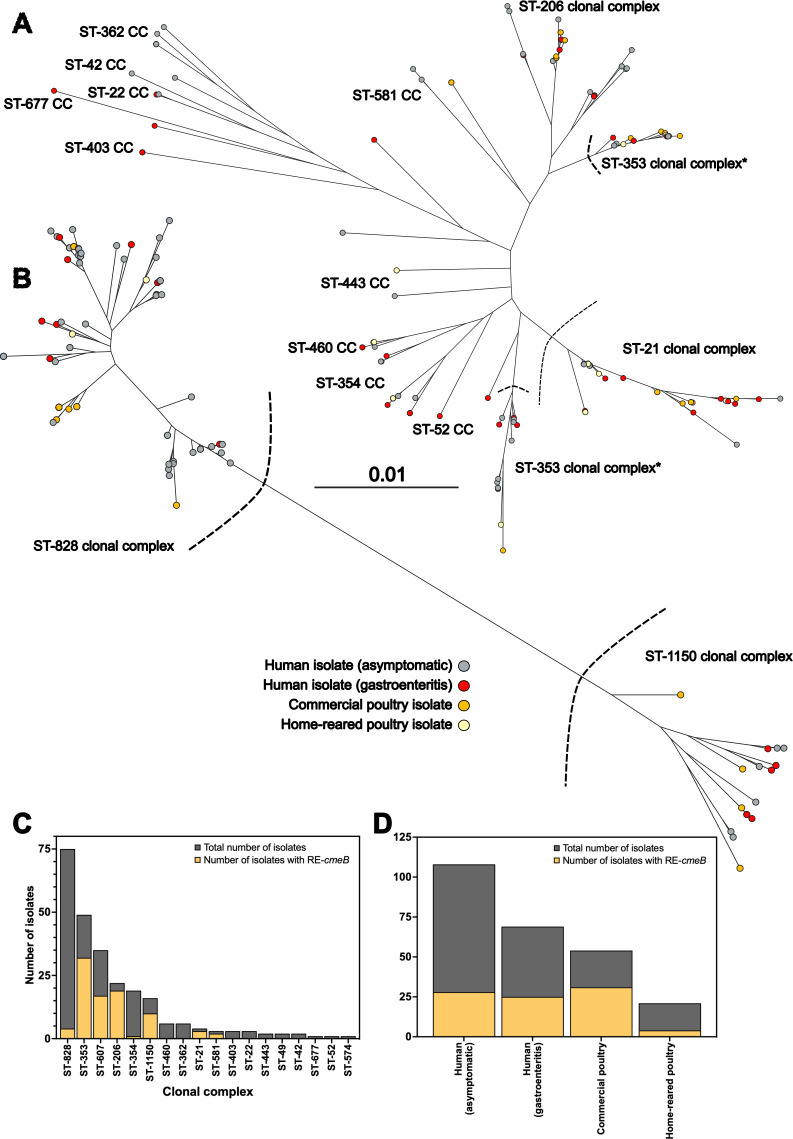
Spread of high resistance RE-cmeRABC genotypes among *Campylobacter* isolates from children and chicken in Peru. Maximum likelihood phylogeny based on the 468 core genes shared among all 252 isolates, visualized separately for *C. jejuni* (**A**) and *C. coli* (**B**). Major lineages are labeled (ST-clonal complexes). Leaves colored by source; isolates from asymptomatic human carriage (gray), gastroenteritis cases (red), commercial poultry (orange), and home-reared poultry (yellow). Tree scale indicated as percentage of total alignment (412,566 bp), and all branches are supported by more than 1,000 bootstraps. Dotted lines indicate the threshold between clonal complexes for *C. jejuni* and *C. coli*. Distribution of susceptible and high-resistance *cmeB* alleles (RE-*cmeRABC*-associated alleles are shaded in yellow) among clonal complexes (**C**) and isolate source (**D**).

We constructed a phylogeny by aligning nucleotide sequences from all core genome genes (*n* = 468, present in 99% of isolates). *C. jejuni* (Fig. 1A) and *C. coli* (Fig. 1B) populations were visualized separately and displayed a highly structured population, as previously described (61, 62). We identified 92 different STs (58 *C*. *jejuni* and 34 *C*. *coli*) from 16 different CCs (14 *C*. *jejuni* and 2 *C*. *coli*). Consistent with other studies, the ST-353 CC was not monophyletic, with two main clusters of isolates (63, 64). Isolates that caused gastroenteritis were distributed across the tree, with isolates from human infection or colonization belonging to many of the same CCs as those from chickens (Fig. 1A and B).

Despite clear clustering of lineages in both species, there was little clustering of the RE*-cmeB* genotype, with isolates containing alleles associated with high resistance spread across the tree and not limited to any specific clonal complex (Fig. 1C). A greater proportion of *C. jejuni* isolates carried the RE*-cmeB* genotype (51.9%, 83 of 160), including a high proportion of isolates from CC ST-353 and ST-607. However, none of the 15 isolates from CC ST-354 carried the RE*-cmeB* genotype. Overall, fewer *C. coli* isolates carried the *RE-cmeABC* locus (17.4%, 16 of 92), compared to *C. jejuni* (*X*^2^ [1, *n* = 252] = 29.12; *P* value of <0.001). Most *C. coli* isolates that carried the RE*-cmeABC* genotype were from the highly introgressed ST-1150 CC (43.8%, 7 of 16) (62, 65). Isolates collected from commercial poultry were much more likely to have the RE*-cmeB* genotype (77.8%, 42 of 54) compared to backyard poultry (23.8%, 5 of 21) (*X*^2^ [1, *n* = 75] = 18.8, *P* value of <0.001) (Fig. 1D).

### *cmeB* has co-evolved differently from other *cme* genes

**Fig 2:**
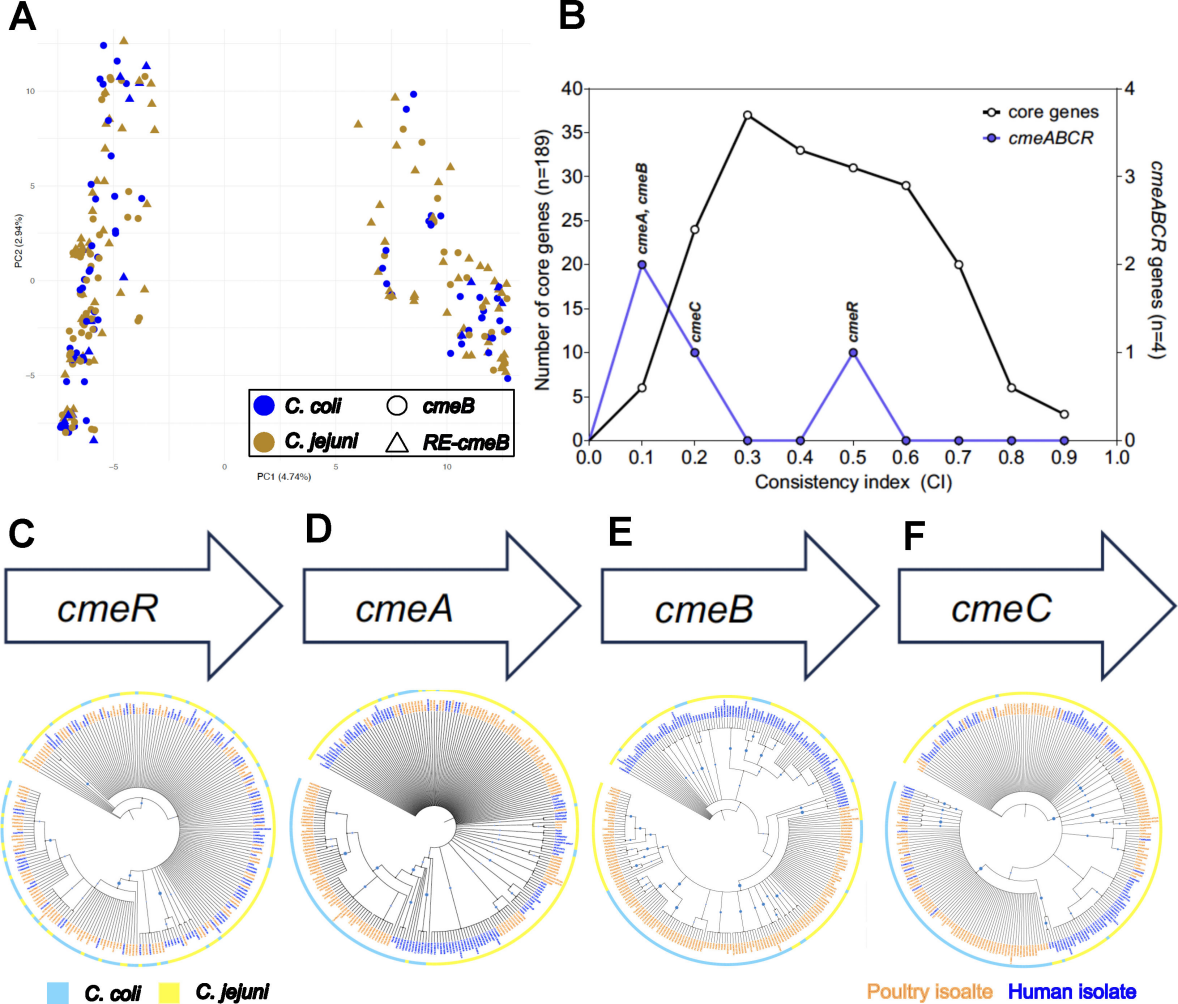
Individual phylogenies of genes from the cmeRABC locus are different. (A) Principal component analysis (PCA) of 252 *Campylobacter* isolates, demonstrating the diversity of the strains based on protein versus protein comparison between each strain. PCA plot generated using the micropan software in R (48). (B) Phylogenies of *cmeA* and *cmeB* showed less congruence with phylogenies of 189 strict core genes. (C–F) Locus map and phylogeneis based on individual protein sequences of the *cmeRABC* locus of *Campylobacter* (C) CmeR, (D) CmeA, (E) CmeB, and (F) CmeC. In all trees, blue coloring of leaf labels indicates that the strain contains a high resistance-associated RE-*cmeB* gene, and orange coloring indicates susceptible-associated *cmeB* genes. The outer ring indicates species designation, with coloring representative of *C. coli* (blue) and *C. jejuni* (yellow) isolates.

A comparison of the isolates (*n* = 252) predicted protein content (protein-by-protein comparison for each predicted protein in the genome of all isolates) using a PCA did not identify any clustering by species or presence of the RE*-cmeB* allele (Fig. 2A). To better understand the ancestry of each individual component of the *cmeRABC* operon, we analyzed each gene independently. There were significantly more alleles (per locus) for *cmeA* and *cmeB*. Calculation of the mean CI, which measures the similarity between the single-gene tree and the tree constructed from the core genome, suggested that *cmeA* and *cmeB* varied most from the core genome phylogeny. Significantly lower CI values were observed among genes in the *cmeRABC* operon (CI 0.202 [±0.193] compared to CI 0.456 [±0.178]; Mann–Whitney test, *U* = 115, *P* value = 0.013) compared with those among 189 published core genes (Fig. 2B). Specifically, the phylogenies constructed from *cmeA* and *cmeB* genes deviated considerably from the core genome phylogeny (CI for *cmeA*: 0.113 and CI for *cmeB*: 0.103, where one is identical to the core genome phylogeny). This is indicative of enhanced horizontal gene transfer–recombination facilitating the movement of these genes in multiple genetic backgrounds.

Single-gene phylogenies were constructed based on the protein sequence of the product for each gene independently (Fig. 2C). CmeR protein coding sequences demonstrated the most sharing between species (Fig. 2D), with the three other proteins rarely shared between the two species. CmeA forms the periplasmic subunit of the pump, and isolates clustered almost completely by species, with subclusters of isolates from poultry and human sources (Fig. 2E). CmeB is the inner membrane subunit, and protein sequences segregated completely by their source (human or poultry), with subclusters of isolates from each species (Fig. 2F). CmeC is the outer membrane subunit of the efflux pump, and a comparison of isolates based on CmeC protein sequences showed segregation almost entirely between species, with some mixing between poultry and clinical isolates (Fig. 2F). Both groups of isolates (RE*-cmeB* and *cmeB*) contain *C. jejuni* and *C. coli* isolates. However, this sharing of alleles between species is more commonly observed in the *cmeB* group (ratio 1:1), compared to the RE*-cmeB* group (ratio 21:4, *C. jejuni* to *C. coli*) (Table S1). Figure S1 is a phylogenetic tree based on the core genome of the 253 strains from the study but also includes those isolates with the RE-*cmeB* genotype and the presence/absence of other major antibiotic resistance genes and/or mutations, including *gyrA* gene mutation, 23S rRNA gene mutation, and *tetO* gene.

We also compared the amino acid sequences of the CmeB and RE*-*CmeB variants. CmeB proteins had amino acid identities between 95% and 100% with other CmeB variants. The difference between *C. jejuni* and *C. coli* was closer to 95%. Protein identities between different RE-CmeB variants were approximately 98%. The sequence identity between CmeB variants and RE*-*CmeB variants ranged from 80.6% to 82.4% (Fig. S2). The percentage of charged amino acids among CmeB was above 18.2%, and the percentage among RE-CmeB was less than 18% (Fig. S3). Although this difference is slight, examination of the aligned protein sequences demonstrates that the positions of charged amino acids are altered between the two groups.

### Macrolide resistance-associated genotypes originate in *C. jejuni*

**Fig 3:**
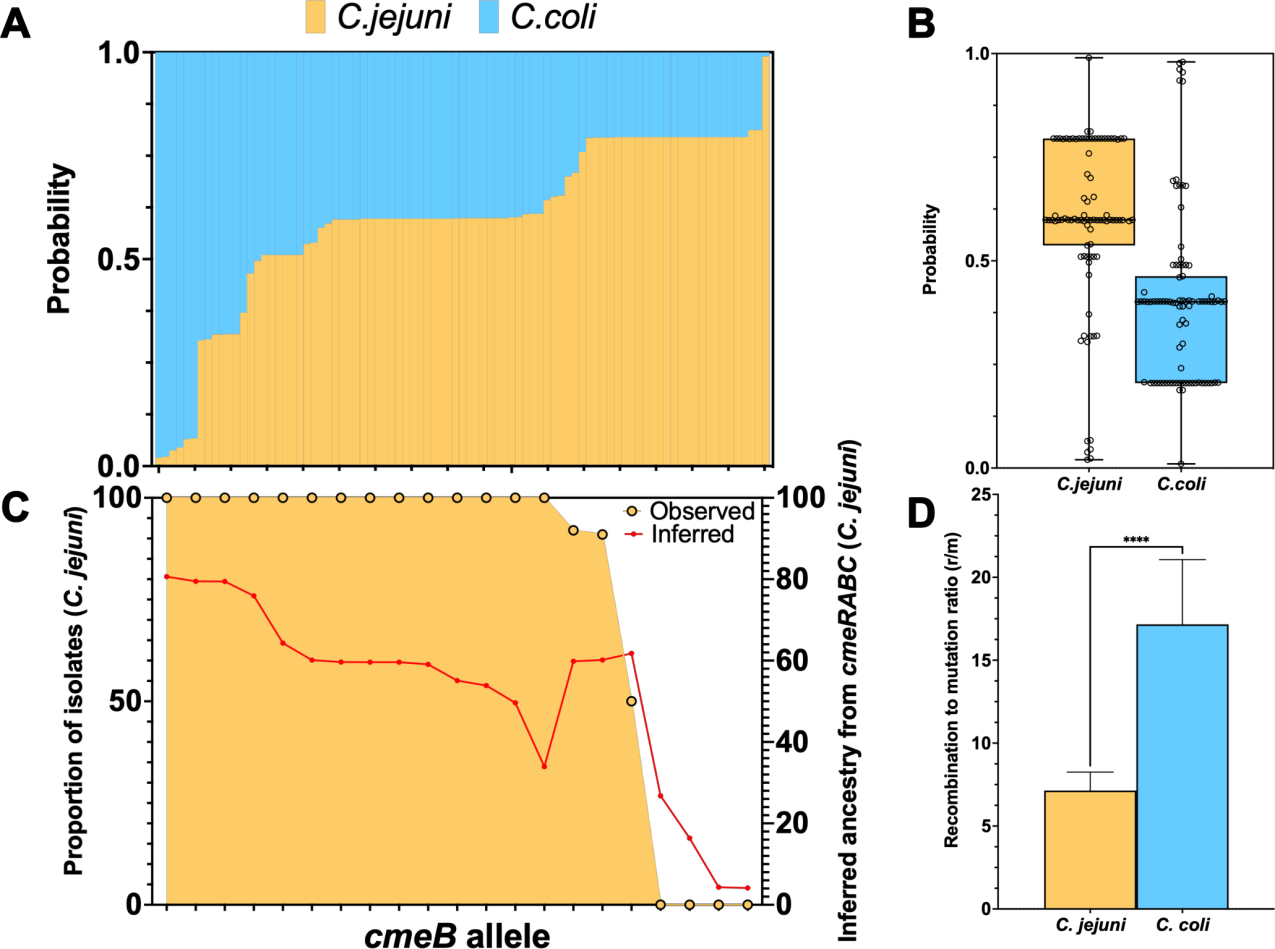
Assigning high resistance-associated RE-*cmeB* alleles to *Campylobacter* spp. (A) Inferred ancestor species of 21 RE-*cmeB* alleles (representing 86 isolates) based on an evolutionary model trained on the four genes of the *cmeRABC* locus, including 56 different *cmeB* alleles that segregated completely between *C. jejuni* (*n* = 32) and *C. coli* (*n* = 24) populations. Self-test accuracy over 95%. (B) Box plot summary of inferred ancestry for all 21 RE-*cmeB* alleles. (C) Comparison of inferred ancestry with the observed origin of all RE-*cmeB* alleles. Three alleles with mixed ancestry, including *cmeB* allele 1640, which was found in an equal number of *C. jejuni* and *C. coli* isolates. (D) Box plot summary of the ratio between recombination and mutation sites, with increased recombination observed in *C. coli*; ****,P value <0.0001.

We further analyzed the *cmeRABC* operon using STRUCTURE, which assigns individuals to source populations. We trained a data set of 136 isolates (71 *C*. *jejuni* and 65 *C*. *coli*, Table S2) to assign species based on the allelic profile of the four genes contained in the *cmeRABC* operon. This included 56 different *cmeB* alleles that segregated completely between *C. jejuni* (*n* = 32) and *C. coli* (*n* = 24) populations. By masking the origin species of a third of isolates in the training data set, we achieved a self-test accuracy of 95.6%. Using this model, we assigned species to isolates demonstrating macrolide resistance to putative source species populations (21 RE-*cmeB* alleles, representing 86 isolates). Most *cmeB* loci could be assigned to populations in *C. jejuni* (Fig. 3A and B). By comparing the inferred species with the observed distribution of isolates from which each RE-*cmeB* allele was found, we can see that most alleles have likely evolved separately in each species; i.e., they were found exclusively in isolates from a single species and had more than 60% inferred ancestry based on the *cmeRABC* locus (14 *C*. *jejuni* and 4 *C*.*coli*, Fig. 3C). The remaining three alleles were found in both *C. jejuni* and *C. coli* isolates, and one allele (allele #1640) was found in an equal number of *C. jejuni* and *C. coli* isolates yet is inferred to originate from *C. jejuni*.

Introgression across the species boundary between *C. jejuni* and *C. coli* has previously been observed, with up to ~20% of the genome able to be shared between species (66). The distribution of RE*-cmeB* genotypes around the tree, among divergent lineages, suggests a role for horizontal gene transfer (HGT) in the emergence of this multidrug phenotype. Estimates of recombination demonstrated higher rates of recombination in *C. coli* compared to *C. jejuni* (Fig. 3D). Per branch estimations of recombination identified not only significant differences between *Campylobacter* spp. (*C. jejuni* mean *r*/*m* = 8.23, *C. coli* mean *r*/*m* = 22.90; Mann–Whitney test, *U* = 19,752, *P* value of <0.001) but also a trend toward elevated rates of recombination in commercial poultry (commercial poultry isolates mean *r*/*m* = 10.60, backyard poultry isolates mean *r*/*m* = 4.13; Mann–Whitney test, *U* = 487, *P* value of 0.426 not significant).

### Global distribution of RE*-cmeB* genotypes

**Fig 4:**
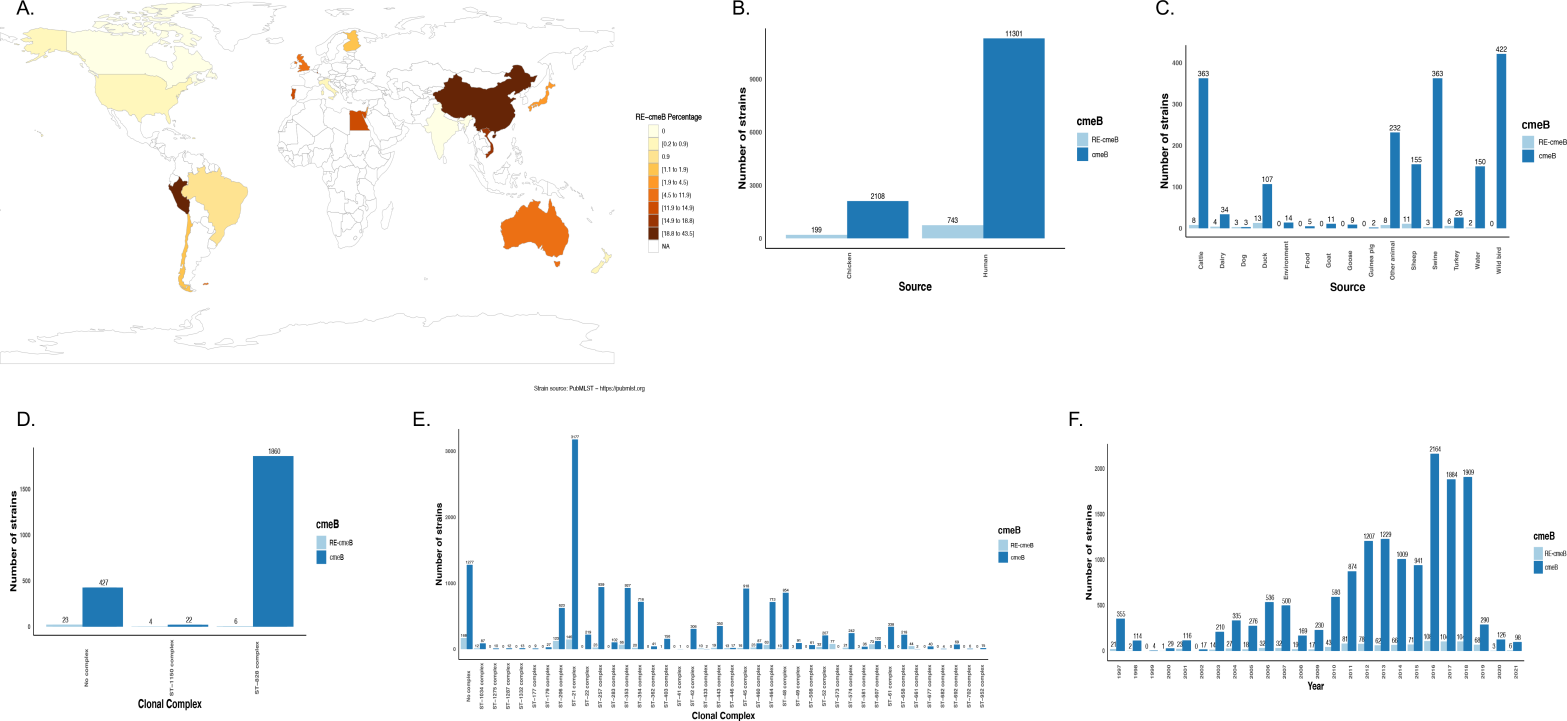
Global, source, sequence type, and time distribution of the high-resistance *cmeB* gene from *Campylobacter* strains in the PubMLST database. (A) Percentage of *Campylobacter* strains from various countries around the world with the RE-*cmeB* high-resistance gene (minimum 40 genomes). (B) Number of *Campylobacter* strains isolated from either chickens or human clinical cases with or without the RE-*cmeB* gene. (C) Number of *Campylobacter* strains isolated from sources other than chickens or human clinical cases with or without the RE-*cmeB* gene. (D) Number of *Campylobacter coli* strains based on clonal complex with or without the RE-*cmeB* gene. (E) Number of *Campylobacter jejuni* strains based on clonal complex with or without the RE-cmeB gene. (F) Number of strains based on year of isolation with or without the RE-*cmeB* gene.

To provide context to our observation that RE*-cmeB* genotypes were found in 39.3% (99 of 252) of all our Peruvian isolates (Table S1), we searched PubMLST database for genomes containing putative RE*-cmeB* genotypes identifying that 6.11% were RE-cmeB (986 of 16,120). It should be noted that available strains were biased toward United Kingdom, New Zealand, and the United States, which together made up 82.6% (13,311 of 16,120) of the available genomes. Matches to the RE*-cmeB* genes (90% similarity over 90% gene length) in isolates from 20 countries and 6 continents identified the highest proportion identified of RE-cmeB from China (43.3%, 20 of 46), followed by Peru (18.8%, 79 of 420), Vietnam (16.8%, 104 of 618), Luxembourg (14.9%, 26 of 175), and Egypt (13.7%, 16 of 117). RE*-cmeB* genotypes were not common in the two countries represented by the most genomes in PubMLST, United States (0.8% of 1,046 isolates) and the United Kingdom (5.8% of 11,846 isolates). Additionally, the high percentage of RE-*cmeB* genotype found in Peru was not common in the two neighboring South American countries of Brazil (0.9%, 1 of 113) and Chile (1.2%, 1 of 83; Fig. 4A) that also had significant genomes available. We also explored how common RE-*cmeB* genotypes were in isolates from human clinical cases (6.2%, 743 of 12,044) and possible source hosts, including the most common cause of human infections, chicken (8.6%, 199 of 2,307; Fig. 4B), dogs (50%, 3 of 6), turkeys (18.8%, 6 of 32), ducks (10.8%, 13 of 120), dairy cattle (10.5%, 4 of 38), and sheep (6.6%, 11 of 166; Fig. 4C).

As we observed in our Peruvian data set, *C. coli* RE-*cmeB* genotypes were most common in the highly introgressed ST-1150 CC (18.2%, 4 of 26) compared to the large generalist ST-828 CC (0.3%, 6 of 1,866) (Fig. 4D). The RE-*cmeB* genotype was identified in several *C. jejuni* CCs, especially those with a close relationship with either poultry or human hosts: a high proportion of isolates from host specialist lineages ST-661 CC (44 of 46 isolates, 95.7%; poultry-associated lineage), ST-446 CC (13 of 17 isolates, 43.3%; human-associated lineage), ST-206 CC (123 of 746 isolates, 16.5%; generalist lineage), ST-464 CC (63 of 776 isolates, 8.1%; poultry-associated lineage), and ST-353 CC (66 of 993 isolates, 6.6%; poultry-associated lineage; Fig. 4E). The RE-*cmeB* genotype was not identified in ruminant-associated lineages, such as ST-42 CC and ST-22 CC, or the wild bird-associated lineage, such as ST-658. The earliest identifiable isolate date in the PubMLST database was 1997 and 5.6% of isolates (21 of 376) collected that year contained the RE-*cmeB* genotype (Fig. 4F). RE-*cmeB* genotypes have become increasingly common in isolates collected more recently. There was limited identification of RE-*cmeB* genotypes in isolates from 1998 to 2003 (0%–6.3%), but between 2003 and 2018, prevalence increased slowly to 10.1%. There is a sharp spike in prevalence to 19.0% (68 of 358) in 2019, which may be biased by the low number of genome submissions.

## DISCUSSION

Despite the global predominance of *Campylobacter* as a cause of bacterial enteritis, genomic epidemiology has been highly focused in high-income countries. We studied a highly resistant strain from the Peruvian Amazon derived from children under the age of 5, industrial poultry, and backyard poultry. Through whole-genome sequencing, we characterized individual genes within the *cmeRABC* locus, which is often associated with increased resistance to multiple antibiotics through changes in an RND efflux pump. Livestock animals are hypothesized to be the primary source of human *Campylobacter* infection in most epidemiological contexts (33, 67), and isolates from both symptomatic and asymptotic human infections clustered alongside isolates from poultry in our phylogeny. Isolates with high resistance-associated genotypes (RE-*cmeB*) were found across multiple genetic backgrounds, and high-resistance genotypes were identified in both human and poultry isolates, although they were significantly more frequent in commercial poultry than in backyard chickens.

Several *cmeB* alleles are shared between *C. jejuni* and *C. coli*. Previous studies have noted extensive introgression from *C. jejuni* to specific clades of *C. coli*, including alleles of the *cmeB* gene (62). Shared *cmeB* alleles in isolates located from distant genetic backgrounds or lineages are consistent with HGT, and we find evidence of increased recombination rates in both *C. coli* isolates and *C. jejuni* isolates from commercial poultry. Recent niche expansion, resulting from industrialization of agricultural methods, may have reduced the barriers to recombination between species (61). While incongruous use of antibiotics in agriculture may have contributed to the selection of high-resistance *cmeB* alleles, relatively few isolates from backyard poultry also carried this genotype. Small-scale breeders and individual chicken owners also have unrestricted access to antimicrobials in the settling from which our samples were collected, and an alternative explanation for the local spread of these resistance phenotypes may be a consequence of bystander resistance. Additionally, small-scale breeders might not use antibiotics as systematically as commercial agriculture, ameliorating the effects of consistent exposure on isolates from backyard poultry compared to commercial poultry. Genes or genotypes can contribute to more than one phenotype, and changes in the *cmeRABC* locus have also been shown to affect resistance to cleaning detergents (that may be used in commercial poultry production) or bile acids during chicken colonization (68–70). Phenotypic cross-resistance may be an indirect consequence of genome evolution in this locus, in which evidence comes from the variability of genes and single nucleotide polymorphisms (SNPs that contribute to changes in resistance. Stepwise increases in the level of resistance conferred by changes in the network of genome elements, including nucleotide changes in the *cmeB* gene, upstream regulator sequence, and additional linked changes in *gyrA* or 23S rRNA genes, suggest that antimicrobial resistance is not the primary phenotype under selection (27). The highly heterogenous prevalence of this important genomic determinant of resistance warrants further investigation. It should be noted that unlike gyrA mutations, which are neutral to *Campylobacter* fitness, the retention of RE-*cmeABC* in populations is likely to be very costly in terms of fitness as this is an ATP-dependent pump.

Our findings hint at a broader, widespread acquisition of MDR elements that can compromise oral antibiotic treatment, particularly in low-resource settings where there is the greatest need for effective antimicrobial treatment. We also highlight the need to incorporate more subtle gene mutations into *in silico* databases for identification (and prediction) of AMR profiles, as allele variation in the *cmeRABC* locus is not currently incorporated into *in silico* AMR determinant identification methods (49, 50, 71). Understanding the mutations that can affect phenotypic resistance is fundamental to a better understanding of the spread of resistance. Mutations in *gyrA* and 23S rRNA echo concerns about the impact of poultry reservoirs on human health and the association between poultry consumption and increased *Campylobacter*-related infections (72, 73). This study provides a snapshot of the distribution of AMR and RE-*cmeB* alleles in Peru. However, by leveraging global collections of *Campylobacter* genomes (via PubMLST), our genomic analysis identifies similar resistance-related profiles in other poultry-associated *Campylobacter* genomes. Providing a global perspective on the prevalence of RE-*cmeB* alleles emphasizes differences in regional dynamics, showcasing substantial variability across countries. Clonal complex associations, particularly with ST-353 and ST-661, underline the role of specific complexes in driving AMR dissemination (64, 74, 75).

While our work provides a snapshot of the current spread of AMR in Peru, improved global sampling efforts help provide context to our findings. Improved genomic epidemiology and pathogen surveillance from a broader range of countries and continents will provide a greater understanding of the evolution and spread of AMR in *Campylobacter*. While recent drives to expand sampling efforts may be contributing to this perceived increase, the lack of genomes deposited from high-income countries, with active pathogen surveillance programs for *Campylobacter,* is striking. There is a lack of current studies that evaluate global genome collections alongside phenotypic antimicrobial resistance profiles. Collections of concomitantly isolated *Campylobacter* isolates from humans, farm-raised and household-raised poultry, as well as other animal sources, are severely limited. This type of study will be essential in efforts to better understand disease transmission dynamics and the spread of AMR, and/or virulence genes. Despite these limitations, we identify enhanced resistance-associated alleles in genomes deposited in PubMLST from around the world, more frequently in recently sampled poultry *C. jejuni* isolates from Peru.

## SUPPLEMENTAL MATERIAL

Figure S1 mbio.02054-24-s0001.pdf Maximum-likelihood tree based on the core genomes of the 253 *Campylobacter* isolates.

Figure S2 mbio.02054-24-s0002.pdf Amino acid identity.

Figure S3 mbio.02054-24-s0003.pdf Charged amino acids.

Table S1 mbio.02054-24-s0004.xlsx *Campylobacter jejuni* and *Campylobacter coli* genome information, antimicrobial resistance phenotypes, genes, and mutations.

Table S2 mbio.02054-24-s0005.xlsx Training data set used for STRUCTURE analysis.

Table S3 mbio.02054-24-s0006.xlsx Recombination parameters as calculated by Gubbins.

ASM does not own the copyrights to Supplemental Material that may be linked to, or accessed through, an article. The authors have granted ASM a non-exclusive, world-wide license to publish the Supplemental Material files. Please contact the corresponding author directly for reuse.
