# Supplementary material for: Sharing of cmeRABC alleles between C. coli and C. jejuni associated with extensive drug resistance in Campylobacter isolates from infants and poultry in the Peruvian Amazon
Source: mBio. 2024 Dec 27;16(2):e02054-24. doi: 10.1128/mbio.02054-24 (PMC11796421; doi:10.1128/mbio.02054-24)
Supplement: Figure S1 — Maximum-likelihood tree based on the core genomes of the 253 Campylobacter isolates. [file mbio.02054-24-s0001.pdf]

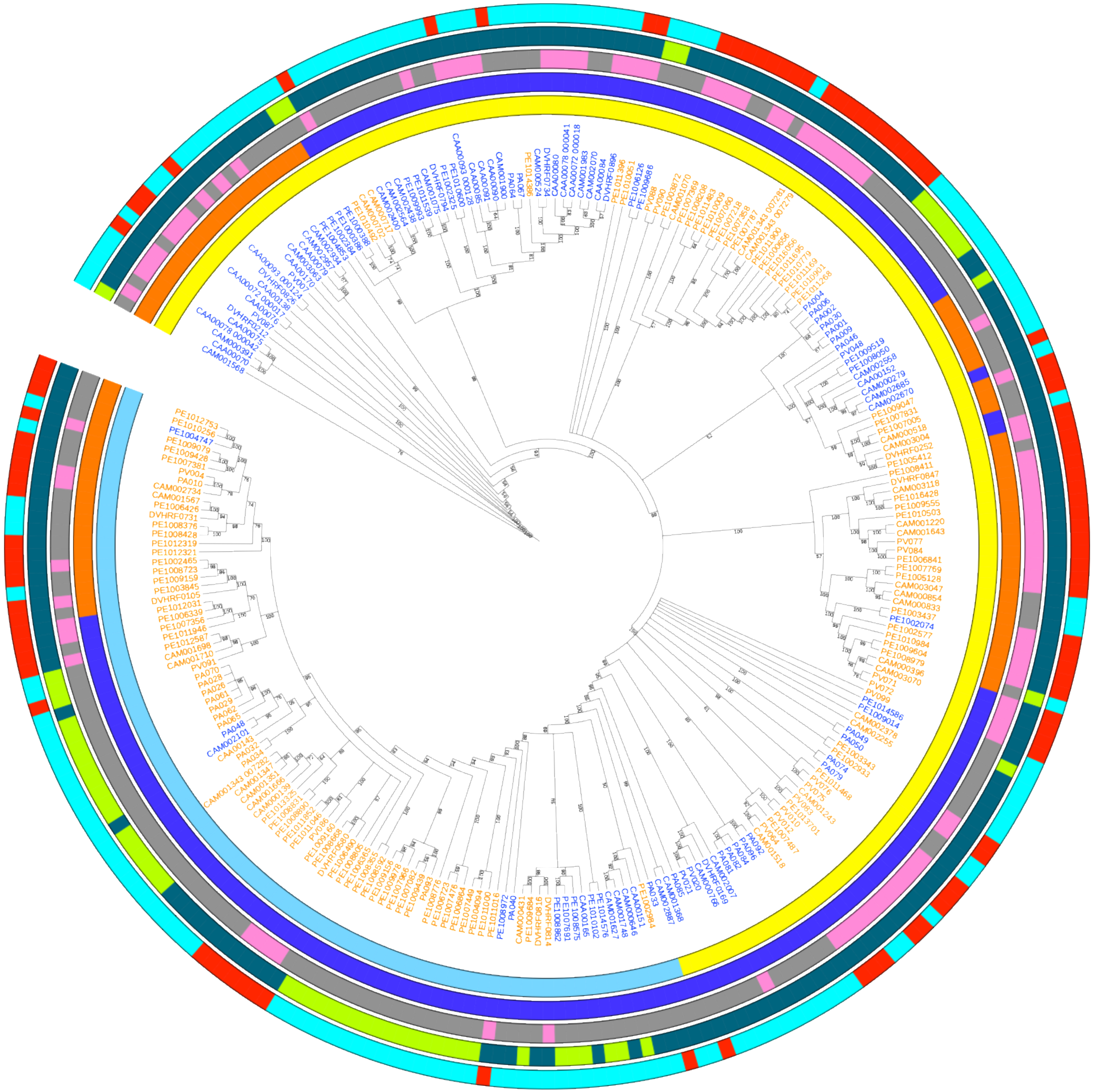

Supplemental Figure 1. Maximum-likelihood tree based on the core genomes of the 253 *Campylobacter* isolates from this study that were bootstrapped 1,000 times. Color of the leaves is based on presence/absence of RE-*cmeB* genotype, orange represents isolates with *cmeB* genotype and blue represents isolates with RE-*cmeB* genotype. Rings represent different metadata about each isolate, inner from outer rings: inner ring – *Campylobacter* species, yellow represents *C. jejuni* and light blue represents *C. coli*; 2nd ring – Source of isolate, orange represents poultry and blue represents clinical; 3rd ring – *gyrA* mutations, silver represents *gyrA* Th186L mutation and pink represents normal *gyrA* gene; 4th ring – 23S rRNA mutations, dark teal represents normal 23S rRNA gene and yellow represents 23S rRNA A2075G mutation; 5th (outer) ring – presence of *tetO* gene, turquoise represents *tetO* gene and red represents absence of gene. The antibiotic resistance genes *floR, fexA, fexB, cfr, optrA,* and *ermB* were not found in any of the isolates. All antibiotic resistance genes present in the isolates are listed in the Supplemental Table 1.
