# Supplementary material for: Sharing of cmeRABC alleles between C. coli and C. jejuni associated with extensive drug resistance in Campylobacter isolates from infants and poultry in the Peruvian Amazon
Source: mBio. 2024 Dec 27;16(2):e02054-24. doi: 10.1128/mbio.02054-24 (PMC11796421; doi:10.1128/mbio.02054-24)
Supplement: Figure S2 — Amino acid identity. [file mbio.02054-24-s0002.pdf]

	PE1009555 -CmeB	PE1010009 CmeB	CAM000717-003781 CmeB	CAM003004 - CmeB	PE1010256 CmeB	PE1009978 -CmeB	PE1009686 -RE-CmeB	PE1009014 RE-CmeB	PE1008575 RE-CmeB
Cj-PE1009555 CmeB	100	98.65	98.85	99.52	97.79	96.35	81.35	80.87	82.4
Cj-PE1010009 CmeB	98.65	100	99.42	98.75	97.12	95.96	81.15	80.67	82.21
CAM000717-003781 CmeB	98.85	99.42	100	98.94	97.02	95.96	81.15	80.67	82.21
CAM003004 - CmeB	99.52	98.75	98.94	100	97.69	96.25	81.35	80.87	82.4
Cc-PE1010256 CmeB	97.79	97.12	97.02	97.69	100	95.1	81.25	80.58	81.92
Cc-PE1009978 -CmeB	96.35	95.96	95.96	96.25	95.1	100	80.87	81.15	81.44
Cj-PE1009686 RE-CmeB	81.35	81.15	81.15	81.35	81.25	80.87	100	98.65	98.85
Cj-PE1009014 RE-CmeB	80.87	80.67	80.67	80.87	80.58	81.15	98.65	100	98.27
Cc-PE1008575 RE-CmeB	82.4	82.21	82.21	82.4	81.92	81.44	98.85	98.27	100
