# Supplementary figures and images for: Sharing of cmeRABC alleles between C. coli and C. jejuni associated with extensive drug resistance in Campylobacter isolates from infants and poultry in the Peruvian Amazon
Source: mBio. 2024 Dec 27;16(2):e02054-24. doi: 10.1128/mbio.02054-24 (PMC11796421; doi:10.1128/mbio.02054-24)

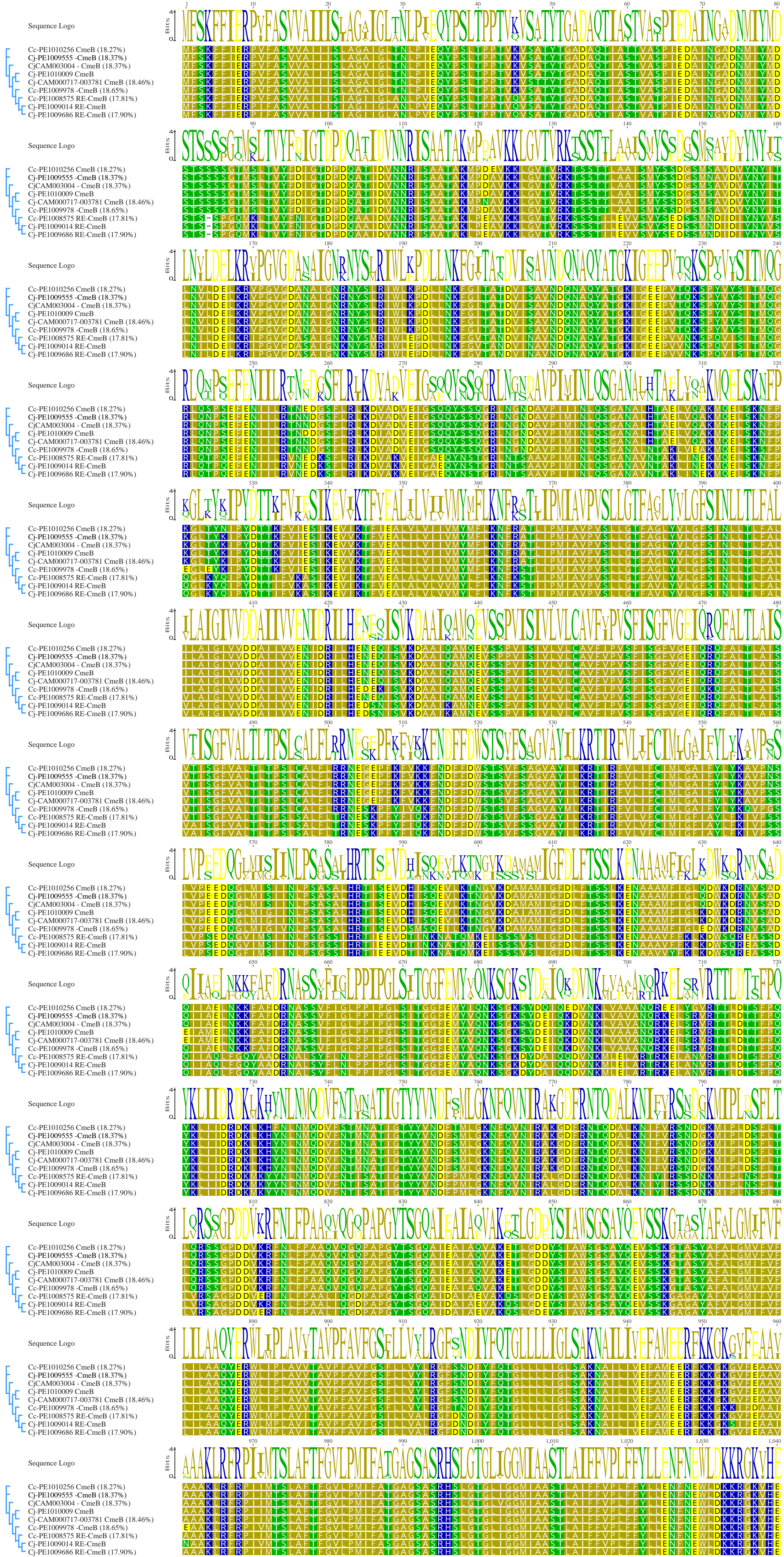

Supplement: Figure S3 — Charged amino acids. [file mbio.02054-24-s0003.pdf]
